# In this issue

**Published:** 2022-09

Venous thromboembolism in COVID-19.* A meta-summary of cases*

ALGhasab et al summarize cases of VTE, including pulmonary embolism, and deep vein thrombosis among COVID-19 patients and discuss their symptoms, diagnostic method, clinical features, and prognosis. A total of 233 articles were identified, 22 describing 48 patients were included. Most patients were men, with a mean age of 56 years. Comorbidities were present in 70.8%, and 85.4% had at least one risk factor of VTE. 56.3% had received anticoagulation therapy. Complications occurred in 27.1% of the patients, and recovery was achieved in 80.4%. They concluded that VTE must be suspected even in patients who had received prior anticoagulant regimens or in stable cases, especially in males, the elderly, and patients with comorbidities and high D-dimer levels.

*see page 979*

