# Supplementary material for: In this issue
Source: Saudi Med J. 2022 Sep;43(9):977. (PMC9987661)
Supplement: Related texts

# Abstract: Original Articles

**Seroprevalence of community-acquired atypical bacterial pneumonia among adult COVID-19 patients from a single center in Al Madinah Al Munawarah, Saudi Arabia.* A retrospective cohort study***

**Figure:** Prevalence of sero-positivity for community acquired atypical pneumonia bacteria co-infections amongst 189 SARS-CoV-2 patients

Alhoufie et al investigate the seroprevalence of the community-acquired bacterial that causes atypical pneumonia among confirmed SARS-COV-2 patients. In this cohort study, they retrospectively investigate the seroprevalence of *Chlamydia pneumoniae*, *Mycoplasma pneumoniae*, and *Legionella pneumophila* among randomly selected 189 confirmed COVID-19 patients at their time of hospital presentation via commercial IgM antibodies against these bacteria. They also carried out quantitative measurements of procalcitonin in patients’ serum. They concluded that their study documented the seroprevalence of community-acquired bacteria co-infection among COVID-19 patients. Procalcitonin was an inconclusive biomarker for non-severe bacterial co-infections among COVID-19 patients. Consideration and proper detection of community-acquired bacterial co-infection may minimize misdiagnosis during the current pandemic and positively reflect disease management and prognosis.

*see page 1000*

**Epicardial adipose tissue thickness and growth differentiation factor 15 in axial spondyloarthritis.* A cross-sectional study***

Okçu et al investigate GDF-15 levels and the thickness of EAT in patients with axSpA and to evaluate their relationship with functional status, disease activity, disease duration, and the type of medical treatment received by the patients. This cross-sectional study is carried out at Kırşehir Ahievran University School of Medicine between February and June 2020. Twenty-nine healthy controls and 44 patients with axSpA were included in the study. They concluded that EAT thickness values were found to be significantly higher in the axSpA group. In addition, GDF-15 was positively correlated with age, Bath Ankylosing Spondylitis Functional Index score, and disease duration.

*see page 1020*

# Abstract: Case Report

**Cardiac myxoma presenting with multisystem involvement**

**Figure:** Brain magnetic resonance imaging

Ageely et al present A 73-year-old Asian male presented with acute left-side weakness, slurred speech, gait imbalance, and subacute constitutional symptoms. Left atrial myxoma was discovered by computed tomography and confirmed by echocardiography. Brain imaging revealed pituitary macroadenoma with subarachnoid and intraventricular hemorrages. The hormonal profile confirmed pituitary apoplexy, for which hormone replacement was initiated. Workup also revealed multiple endocrine tumors and excluded infection and malignancy. Myxoma resection could not be carried out, due to the patient’s rapid clinical deterioration and death. Furthermore, the presence of cardiac myxoma, non-functioning pituitary macroadenomas, and pituitary apoplexy is extremely rare and rarely documented in the literature. Therefore, they emphasize clinical awareness of rare conditions with atypical presentations to improve outcomes.

*see page 1057*
